# The Oculus Rift: a cost-effective tool for studying visual-vestibular interactions in self-motion perception

**DOI:** 10.3389/fpsyg.2015.00248

**Published:** 2015-03-13

**Authors:** Juno Kim, Charles Y. L. Chung, Shinji Nakamura, Stephen Palmisano, Sieu K. Khuu

**Affiliations:** ^1^School of Optometry and Vision Science, The University of New South Wales, Kensington, NSW, Australia; ^2^Department of Child Development, Nihon Fukushi University, Nagoya, Japan; ^3^School of Psychology, University of Wollongong, Wollongong, NSW, Australia

**Keywords:** visual perception, vection, Oculus Rift, self-motion perception, virtual reality

## Abstract

For years now, virtual reality devices have been applied in the field of vision science in an attempt to improve our understanding of perceptual principles underlying the experience of self-motion. Some of this research has been concerned with exploring factors involved in the visually-induced illusory perception of self-motion, known as vection. We examined the usefulness of the cost-effective Oculus Rift in generating vection in seated observers. This device has the capacity to display optic flow in world coordinates by compensating for tracked changes in 3D head orientation. We measured vection strength in three conditions of visual compensation for head movement: compensated, uncompensated, and inversely compensated. During presentation of optic flow, the observer was instructed to make periodic head oscillations (±22° horizontal excursions at approximately 0.53 Hz). We found that vection was best in the compensated condition, and was weakest in the inversely compensated condition. Surprisingly, vection was always better in passive viewing conditions, compared with conditions where active head rotations were performed. These findings suggest that vection is highly dependent on interactions between visual, vestibular and proprioceptive information, and may be highly sensitive to limitations of temporal lag in visual-vestibular coupling using this system.

## INTRODUCTION

Since the modern age of computing there has been a surge of innovation in engaging technologies that have culminated in the form of modern virtual reality devices. From a computer science perspective, “[a] display connected to a digital computer gives us a chance to gain familiarity with concepts not realizable in the physical world. It is a looking glass into a mathematical wonderland” (Sutherland, 1965). The recent release of the Oculus Rift virtual reality headset (http://www.oculusvr.com/) has attracted a surge of interest in the gaming community because it provides cost-effective consumer access to virtual reality technologies that were once unattainable due to expense in construction. However, does the Rift provide the ability for scientists to study the basis of self-motion perception? Here, we examine the usefulness of the Rift in probing some of the visual-vestibular (and also proprioceptive) interactions known to be involved in the perception of self-motion.

Virtual reality displays recreate patterns of optic flow generated by the relative image motion of objects that occurs when we move through the world. Optic flow provides information that is sufficient for us to estimate our angular and linear changes in head position/orientation in six-degrees of freedom (Gibson, 1950). These patterns are also known to generate vection—the illusory perception of self-motion that occurs in completely stationary observers who view these displays (Fischer and Kornmüller, 1930).

Vection was believed to depend on the consistency between visual information concerning self-motion and other available multisensory stimulation, including vestibular information and proprioception. This belief was maintained in the work of Zacharias and Young (1981), who argued that vection should be greatest when visual motion presented to stationary observers simulates constant velocity self-motion; the vestibular organs sense changes in gravitation force applied to the head (i.e., angular or linear accelerations), and remain inactive during prolonged self-motion at a constant linear velocity.

Contrary to the view that multisensory compatibility is necessary for vection, Palmisano et al. (2000) showed that vection can be enhanced when optic flow consistent with linear head acceleration is presented to stationary observers. They added horizontal and vertical head translations of random amplitudes to optic flow simulating self-motion in depth. This simulated random change in coronal head position (or jitter) was found to enhance the strength of vection generated by expanding optic flow simulating self-motion in depth (see also Nakamura, 2012). This vection increase was also observed when adding sinusoidal changes in simulated linear head position to optic flow simulating self-motion in depth (Kim and Palmisano, 2008; Palmisano et al., 2008, 2011; Seno et al., 2011; Kim and Khuu, 2014).

Although perceived depth from motion parallax is one potential explanation for these vection increases observed when adding inconsistent visual acceleration, this does not appear to explain the entire effect. Palmisano and Chan (2004) found that the perceived distance into the display that the observers traveled was not affected by the addition of lateral changes in simulated head position. Also, Kim et al. (2012) found that vection was increased when simulated angular head rotations—which do not constitute a translation of the head—were added to radial flow simulating self-motion in depth. Vection has also been shown to increase with the addition of angular oscillation around the focus of expansion (Kim and Khuu, 2014), which had the added advantage of preserving gaze relative to the center of the display. Note that because only rotations are added to radially expanding optic flow, these displays eliminated the effects of motion parallax present in past studies with linear head movements.

Although the aforementioned studies generated optic flow using large wall-mounted external displays, their geometry may not be consistent with changes in visual perspective during real head movements. The perspective of the display in those studies was not precisely matched to the observer’s vantage point (since the display was fixed to the wall and not to the head of the rotating observer). The angular rate sensors embedded in head-mounted displays (HMDs) eliminate these possible artifacts and provide the functionality to test whether these angular viewpoint changes *per se* enhance vection in depth. However, HMDs also have their own potential limitations.

Riecke and Schulte-Pelkum (2013) have noted several potential limitations of traditional HMDs that can affect vection, including field of view (FoV) and temporal display lag. Previous research has found that even moderate-sized FoV HMDs (52°) can reduce the perceived speed of self-motion in depth, relative to the true speed of self-motion. This perceptual underestimation appears to be due to the dependence of speed perception on lamellar flow at wide visual angles (Banton et al., 2005). HMDs also introduce an inherent temporal phase lag between visual motion and any head motion which might generate internal conflict that could impair vection. Previous research with wall-mounted displays has shown that small phase offsets generated by system lag of approximately 40°^1^ could influence vection (Ash et al., 2011a). That research found that increasing the temporal phase lag of visual display oscillation (beyond 120 ms) generated by linear head oscillation could impair vection. It is likely that similar effects of temporal lag would affect vection in HMD systems.

The Oculus Rift offers some advantages over traditional HMDs. First, the FoV is double that used in the previous Banton et al. (2005) study and far larger than other conventional systems. Second, inbuilt rate sensors register changes in angular head orientation in three dimensions with latencies better than 50 ms (LaValle et al., 2014). Third, the Rift is also packaged with a development kit that allows researchers to rapidly implement displays that can be synchronized with head movements. However, the temporal accuracy to which this can be achieved is likely to be limited by temporal latency (and positional phase lag) inherent in all HMD systems, including the Oculus Rift.

Due to the potential effects of temporal latency on vection (e.g., Ash et al., 2011a), it is important to consider evidence for the effects of phase lag more generally on vection. Effects of phase lag on vection have been observed when imposing large phase offsets in visual feedback during head movement directly. Ash et al. (2011b) found a significant increase in vection strength for contralateral display oscillation synchronized with (the opposite direction of) lateral head translations, compared with an unsynchronized radial flow control. An earlier study found that vection onsets were observed to decline when visual perspective was synchronized with the opposite direction of lateral head translation (Kim and Palmisano, 2010). These studies have used only linear head oscillation. It remains unclear to what extent positional and temporal phase offsets of display movement during angular head rotations may influence vection.

The current study examined (for the first time) the effects on vection in depth of synchronizing the observer’s own angular head oscillations with their visual display motion. The Oculus Rift was used to ensure that display oscillation was more perspectively correct across angular changes in observer head motion. We tested whether multisensory compatibility between visual displays of optic flow and active angular head rotations might also enhance vection in depth. To this end, we also estimated the end-to-end temporal lag of our system from the onset of any head motion to the time that the scene was updated in the visual display.

## MATERIALS AND METHODS

### OBSERVERS

Eight adult observers participated in the study, six of whom were recruited from the University of New South Wales, while the remaining two were recruited from the University of Wollongong. All participants had normal or corrected-to-normal visual acuity and no knowledge of prior vestibular dysfunction. One observer had a peripheral field defect, but this did not significantly affect their experience of self-motion perception (including perceived heading). Procedures were approved by the Human Research Ethics Association (HREA) at The University of New South Wales.

### GENERATION OF VISUAL DISPLAYS

Displays simulated forward self-motion in depth, while either facing forward (purely radial optic flow) or repeatedly rotating one’s head left and right (oscillating radial optic flow). These simulations of optic flow were generated on the Oculus Rift (Version 1.0). This system had a FoV of approximately 110° diagonal. The Rift’s viewing lenses were adjusted to correct for any refractive errors of each participant. A spherical cloud was simulated around the observer by populating the virtual environment (approximately 3 m radius) with 163, 840 blue squares (ranging in optical size from 0.25 to 2.5° with proximity to the observer). The luminance of the dots was 3.5 cd/m^2^ against a black background of 0.11 cd/m^2^. Although the squares loomed in size, their structure was egocentric, such that the aspect ratio and uprightness of each square was held constant with changes in its position across the visual field. The Rift’s FoV made visible approximately 20% of the total number of squares in the environment on any given frame.

### RECORDING ANGULAR HEAD ROTATION

Angular changes in head orientation were recorded using the Rift’s inherent accelerometers and gyros. The Rift contains a three-axis rate sensor to measure changes in angular head orientation around the vertical (yaw), inter-aural (pitch), and naso-occipital axes (roll). Yaw, pitch, and roll changes in head orientation were computed as Euler angles in degrees by a Microsoft Visual Studio 2010 version of the Oculus Rift SDK. Data values were logged to ASCII file at a rate of 10 Hz. The angular values were used to adjust the virtual head orientation simulated by the display using the inbuilt rotation matrix computations of the standard OpenGL pipeline. The update rate for changing simulated head orientation was in the order of 80 Hz, which generated smooth motion of the optic array with almost no perceptible display lag during slow rates of head oscillation. A short period of pre-training ensured the observer’s self-generated head oscillations were targeted at ∼0.5 Hz with amplitudes of approximately 30°. The rate and amplitude was guided by the experimenter for several seconds, before the observer practiced performing them several times at their comfort. We observed visually that the observers tended to oscillate slightly faster over slightly smaller amplitudes after commencing the experiment. The default setting of the Rift ensured that the simulated location of targets in the display compensated directly for angular changes in head orientation. This had the effect of simulating the appearance of objects that were close to stationary in world coordinates.

We also computed the display lag for updating angular changes in the visual scene from the start of a head rotation. This was done using an optical technique to determine the cumulative latency of the sensors in the Oculus to register an angular head rotation and subsequently update visual content in the display. We mounted a 120 fps digital camera on a table to view the surface of the Oculus display with intervening internal optical lenses removed. The Oculus was used to generate two dark spots against a white background. The first spot was completely stationary and would therefore move with the Oculus as it was rotated in front of the camera. The second spot was initially centered in the display, but its vertical position was dictated by the sensed yaw orientation of the Oculus. Custom image analysis software developed previously was used to determine the horizontal and vertical positions of the two spots in degrees (Kim, 2004). We cross-correlated the horizontal position of the stationary spot with the vertically-synchronized spot to estimate the phase lag of the system. This measurement was also performed with the addition of all 163, 840 display dots present as in the psychophysical experiment to account for rendering time. These dots were set to white (i.e., the background color) to prevent interference during video tracking of the two dark spots.

### PROCEDURE

**FIGURE 1:**
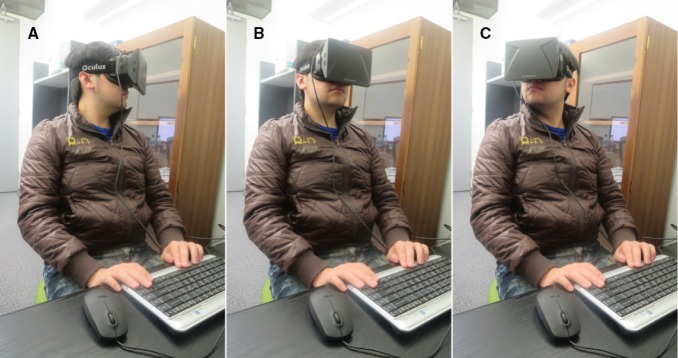
**An observer wearing the Oculus Rift visor demonstrates the full range of head movements within the yaw plane during the presentation of optic flow in active viewing conditions (A–C).** During passive conditions, the observer sat still with their head in a constant forward orientation in yaw **(B)**. A keyboard within arm’s reach provided a comfortable interface for the observer to make psychophysical responses.

Each participant wore the Rift headset while sitting on a height-adjustable chair that maintained their legs comfortably at close to right angles. The participant initially looked ahead with their head erect in darkness. Sample images of the setup and procedure (the room lights were turned on for these photos to improve visibility) are shown in Figure 1. The experimenter executed the simulation application, after instructing the observer to fixate at the green central target prior to each trial and to reset their gaze position back to the center of the display directly afterward (i.e., before the start of the next trial of optic flow). During each of the displays, the observer was instructed to look at the center of the optic flow pattern and concentrate on the experience (if any) of illusory self-motion in depth. The observer was asked to press the spacebar when vection was first experienced during the trial and then had to provide a vection strength rating at the conclusion of each trial. Thus we were able to obtain measures of both vection onset latency and vection strength for each of the experimental conditions described below.

Participant’s rated vection strength by adjusting a vertical meter ranging from 0 to 100. We instructed observers to set it to 0 if they felt completely stationary the whole time, 100 if they felt self-motion that was indistinguishable from physical self-motion the whole time, and values in between according to strengths ranging between the limits. The experiment involved two blocks of trials of either active or passive yaw display oscillation. The first block of active trials required observers to make yaw-plane head movements at around 0.5 Hz and ±30°. Observers were instructed to keep their head oscillations the same across conditions. There were three conditions that varied the coupling of display changes with angular changes in yaw head orientation: contralateral, ipsilateral, or pure radial flow.

The contralateral condition ensured that display orientation in yaw compensated for head rotation. That is, the focus of expansion moved in the opposite direction to the observer’s yaw head rotation, holding constant the spatiotopic location of flow field. The ipsilateral condition had the opposite effect in yaw, whereby the simulated head orientation in yaw was double the observer’s actual head orientation. This had the effect of causing the focus of expansion to move in the same yaw direction as the head rotation, but by twice the amplitude. In the pure radial flow control, the focus of expansion was always aligned with the yaw orientation of the observer’s nose irrespective of their head orientation. That is, pure radial flow did not use any of the yaw head rotation data to update the display. The second block of passive trials involved observers sitting stationary and watching playbacks of their recorded head movements during the active trials. Both contralateral and ipsilateral playbacks generated the same passive visual stimulation. All participants performed trials with all conditions. Condition order was completely randomized within and across blocks.

The approach of passively playing back visual displays generated by actively moving observers was described by Kim and Palmisano (2008), and has since been successfully used by other researchers (e.g., Seno et al., 2013). Although it is likely that there would be differences in task demand between conditions requiring active head movement and passive viewing, the potential increase in attentional demand of executing head movements does not appear to significantly alter vection. For example, Kim and Palmisano (2008) found no difference in vection between passive viewing conditions and active conditions where inter-aural head translations were made by the observer.

### STATISTICAL ANALYSIS

Vection strength and latency data were analyzed using repeated-measures analysis of variances (ANOVAs) to identify any main and interaction effects of display synchronization and active versus passive viewing. Follow-up pairwise comparisons for mean differences were made using repeated-measures *t*-tests.

## RESULTS

### VECTION STRENGTH RATINGS

**FIGURE 2:**
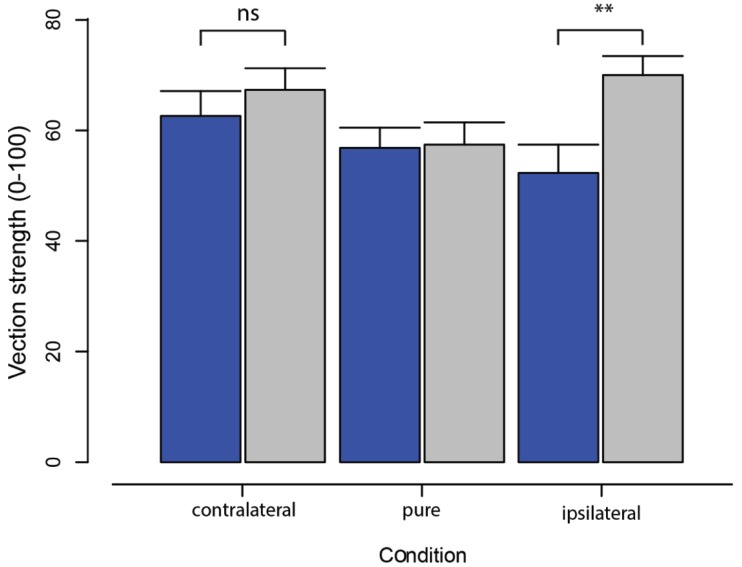
**Mean and standard errors for vection strength ratings obtained in each of the three radial flow display conditions (contralateral, pure, ipsilateral) with either active (blue) or passive (gray) viewing.** Note the larger increase in vection strength during ipsilateral oscillation for passive compared with active viewing of the same visual information. Note that the difference in vection strength between active and passive conditions using ipsilateral synchronization (**) was not significant when using contralateral synchronization (ns).

Bar plots in Figure 2 show the mean vection strength ratings across all observers in the three experimental conditions of display coupling in both active (blue) and passive (gray) viewing conditions. A repeated-measures ANOVA found a main effect between passive and active viewing of optic flow (*F*_1,6_ = 10.39, *p* < 0.05), whereby passive viewing generated greater vection overall than active viewing. Although there was no main effect of oscillation condition (*F*_2,12_ = 3.45, *p* = 0.07), there was a significant two-way interaction effect (*F*_2,12_ = 5.87, *p* < 0.05).

Referring to Figure 2, the interaction effect can be seen in the significant increase in vection strength obtained with passive compared with active viewing of ipsilateral oscillation (*t*_6_ = 4.90, *p* < 0.005), which was not statistically different during viewing of contralateral oscillation (*t*_6_ = 1.09, *p* = 0.32) or pure radial flow (*t*_6_ = 0.16, *p* = 0.88). To further investigate this interaction, we pooled data for the ipsilateral and contralateral conditions for each observer and compared the resulting mean vection strength against that obtained with pure radial flow. Vection strength was found to be significantly greater in the oscillating versus pure radial flow conditions when passively viewing displays (*t*_6_ = 2.56, *p* < 0.05), but not when actively viewing displays (*t*_6_ = 0.39, *p* = 0.71).

### VECTION ONSET LATENCY

**FIGURE 3:**
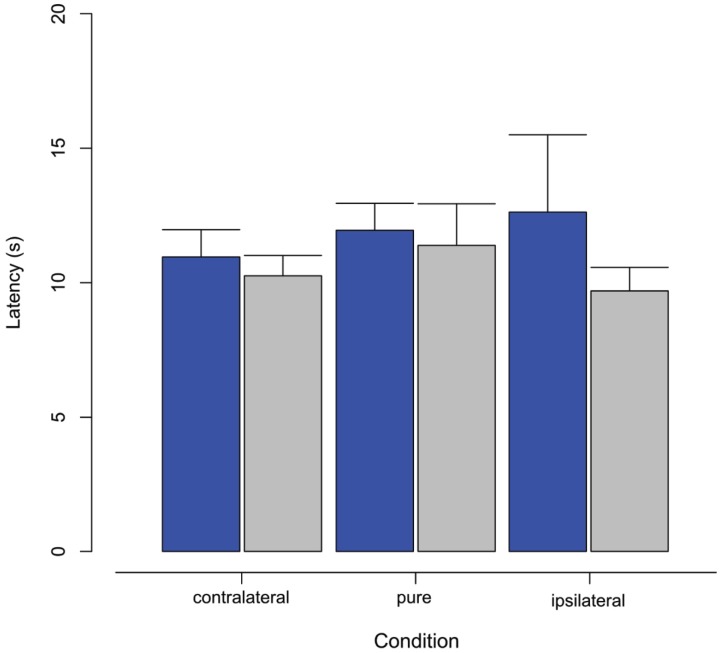
**Means and standard errors for vection onset latencies obtained in each of the three radial flow display conditions (contralateral, pure, ipsilateral) with either active (blue) or passive (gray) viewing**.

Bar plots in Figure 3 show the mean vection onset latencies across all observers in the three experimental conditions of display coupling in both active (blue) and passive (gray) viewing conditions. A repeated-measures ANOVA found no significant main effects of viewing condition (*F*_1,6_ = 0.64, *p* = 0.45) or display oscillation condition (*F*_2,12_ = 0.43, *p* = 0.66). There was also no significant interaction effect (*F*_2,12_ = 0.41, *p* = 0.67). Although there were no significant differences between vection onset latencies, the pattern of differences in the means is inversely consistent with the vection strength ratings.

### ANGULAR HEAD OSCILLATION

**FIGURE 4:**
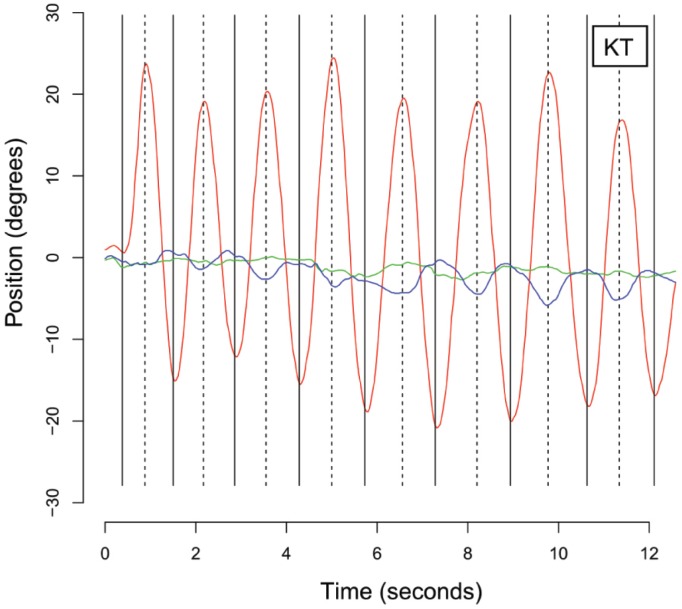
**Raw traces showing yaw (red), pitch (green) and roll (blue) position of the head measured as Euler angles in degrees over time (in seconds) during the first 12 s of a trial performed by a representative naive observer (KT).** Note that the principal direction of head rotation is in yaw. Vertical solid and dashed lines indicate estimated troughs and peaks in the positional amplitude of yaw head rotation.

Traces in Figure 4 show time-series plots of yaw, pitch and roll head orientation for one representative observer on one trial. During active trials, head movements occurred primarily in the yaw plane, corresponding to the instructions given to observers. We first determined the peaks as the turning point from the derivative of angular head orientation (vertical solid and dashed lines). Overall amplitude of excursion of the head in the yaw plane was computed for each observer in each active condition as the mean peak-to-peak amplitude of head rotation. The mean amplitude range of yaw head rotation was 43.6° (SD = 18.0°). A repeated-measures ANOVA found no significant difference in the *amplitude* of yaw head rotation across the three active experimental conditions (*F*_2,12_ = 1.21, *p* = 0.33).

We also computed the mean frequency of head oscillation within the yaw plane, determined to be 0.53 Hz (SD = 0.187 Hz) after computing the overall mean difference between consecutive peaks as well as consecutive troughs in yaw head orientation. A repeated-measures ANOVA found no significant difference in the *frequency* of yaw head rotation across the three active experimental conditions (*F*_2,12_ = 1.33, *p* = 0.30).

The results of our phase lag analysis of the system revealed an end-to-end display lag of 196.7 ms from the time that a head movement was initiated. This lag was obtained when the visual display rendered the same number of dots as presented in during the psychophysical experiment. As a point of reference, the end-to-end lag declined to 37.9 ms when only the two target dots necessary for the measurement was used. This would suggest that much of the lag was due to rendering time in generating the optic flow display in our experiment.

## DISCUSSION

We primarily sought to determine whether the initial release of the Oculus Rift can serve as a viable tool for exploring multisensory interactions involved in the perception of self-motion. We used the Rift to manipulate the level of visual-vestibular (and proprioceptive) conflict in human observers to determine whether such conflicts affect the experience of vection. We expected that synergistic multisensory stimulation would enhance vection strength. In support of this prediction, adding an optic flow component that was contralateral to the observer’s head rotation generated the best vection during active viewing conditions, as indicated by greater vection strength ratings. In comparison, active viewing conditions where the optic flow was independent of the observer’s head-movements (i.e., pure radial flow) generated weaker vection, but this vection was still better on average than that generated by ipsilateral flow condition. Surprisingly however, passive viewing of optic flow generated stronger overall illusions of self-motion than the active viewing conditions.

The current findings provide some support for the view that head-mounted virtual reality offers a portable and practical solution to examining vection and the underlying role of multisensory integration. Our data suggest that vection depends on specific constraints in the compatibility between visual and vestibular sensory signals. Although it was originally thought that visual-vestibular conflict impairs vection (Zacharias and Young, 1981), the work of Palmisano et al. (2000, 2008, 2011) suggested visual information might override vestibular signals or at least dominate the perception of self-motion (see also Nakamura, 2013). This visual dominance may arise because vection strength was improved when passively viewing *linear* viewpoint oscillation while the head was completely stationary. Visual dominance during vection is supported by neurophysiological evidence showing that the vestibular cortex exhibits reduced activity, whereas visual cortical regions increase in activity, during purely visual simulated self-motion (Brandt et al., 1998). Further in contrary to the notion that visual-vestibular conflict impairs vection, we found in the present study that *angular* viewpoint oscillation also increased vection strength, similar to other recent studies (Kim et al., 2012; Kim and Khuu, 2014).

We also found evidence that the Oculus provided sufficient synergistic visual-vestibular information to enhance vection. Specifically, we found that display rotation that compensated for head rotation (contralateral rotation) generated stronger vection than display rotations that did not compensate for head rotation (ipsilateral rotation). This finding with angular oscillation is similar to the finding with linear oscillation of Ash et al. (2011b), where it was found that counter-phase display oscillation significantly improved vection strength. However, the effect in that study was small in extent, consistent with a study by Kim and Palmisano (2010) who found that vection strength was similar during lateral linear head translations, regardless of whether display oscillation was contralateral or ipsilateral to head oscillation. For comparison, we obtained a larger effect here with angular oscillation using the Oculus Rift, which could be attributed to the greater size and geometric accuracy in the presentation of optic flow.

The synergistic visual-vestibular benefits of the Rift were limited when we compared vection strength across active and passive conditions. However, it is possible that the greater strength in vection in passive conditions compared with active conditions is due to temporal visual-vestibular conflict. The end-to-end latency from head rotation to display update was approximately 196.7 ms. This latency appeared to be largely comprised of a delay in rendering time, as the end-to-end latency of updating the display without the presentation of optic flow was approximately 37.9 ms, a value in line with the initial hardware lag from head movement to encoding by inertial MEMS sensors of the Rift (LaValle et al., 2014). Although the total system lag from head movement to display was well above that measured in the Ash et al. (2011a) study (∼113 ms), the implications of these prolonged latency values cannot be reliably inferred at present due to differences in methodology (i.e., linear versus angular). We therefore recommend that future vection studies should consider the advantage of using GPU rendering to improve on the rendering times and temporal latencies that we obtained here.

Unlike Ash et al. (2011a), the display configuration we examined here had a larger FoV (as opposed to smaller FoV) that was head-mounted (as opposed to external) and generated angular display oscillation (as opposed to inter-aural linear display oscillation). The 198 ms end-to-end latency of our system is longer than that of Ash et al. (2011a) raising the possibility that differences between active and passive viewing could be attributed to the inevitable temporal visual-vestibular inconsistency. However, in any event, the differences in angular versus linear head movements across these studies make the data very difficult to compare. Future research would gain greater insight into this possibility by systematically altering the duration of lag between angular head rotation and display changes, while holding all other parameters constant. This could be assessed using any of the emerging brands of head-mounted display technology, including the recent DK2 release and future models of the Oculus Rift. An assessment of this form would determine the critical latency for tolerating lag in the angular head rotation, similar to that determined by Ash et al. (2011a) for the linear head translations.

It is also possible that differences in vection between active and passive viewing could be attributed to variations in perceived path of vection. The end-to-end temporal lag of the system would cause the focus of expansion to vary spatiotopically relative to the true head orientation. This would simulate a change in simulated heading that could have the potential to generate the experience of self-motion along a curvilinear path in depth. However, such perceptual distortions appear to be have been minimal in the current study as no observers indicated significant deviations in vection path from purely linear self-motion in depth.

Based on the findings obtained here, it can be concluded that the use of portable head-mounted virtual reality devices such as the Oculus Rift are of potential use for studying vection, especially in small enclosed spaces and where space is limited. However, as a mode of presenting visual information in an immersive virtual environment, the coupling of visual motion with head motion *per se* did not generate a superiority in visual experience of self-motion perception. It is possible that this might relate to the undesirable temporal lag of the system, which will hopefully be improved in future hardware developments.

It is also unclear to what extent such phase lags might induce motion sickness. Previous research has reported that visually simulated (linear) head oscillation increases motion sickness symptoms during vection (Palmisano et al., 2007). During active viewing conditions (such as those examined here), it would be interesting to determine the effects of phase lag on this oscillation-enhanced motion sickness, as well as on the relationship between vection strength and motion sickness. However, future studies in this area should also consider the possible intervening role of multisensory input from the vestibular system and proprioception, the presence of which might serve to decrease visual dominance in vection in active viewing conditions.

### Conflict of Interest Statement

The authors declare that the research was conducted in the absence of any commercial or financial relationships that could be construed as a potential conflict of interest.

## Footnotes

1 This angular offset was directly computed from the 120 ms lag at a head movement oscillation of 1 Hz in the Ash et al. (2011a) study.
